# Metabolite Profiling and Characterization of LW6, a Novel HIF-1α Inhibitor, as an Antitumor Drug Candidate in Mice

**DOI:** 10.3390/molecules26071951

**Published:** 2021-03-30

**Authors:** Kiho Lee, Ji-Yoon Lee, Kyeong Lee, Cho-Rock Jung, Min-Ju Kim, Jung-Ah Kim, Dong-Gu Yoo, Eun-Jin Shin, Soo-Jin Oh

**Affiliations:** 1 Department of Pharmacy, College of Pharmacy, Korea University, Sejong 30019, Korea; kiholee@korea.ac.kr; 2 Asan Medical Center, Asan Institute for Life Sciences, Seoul 05505, Korea; lsuklee@hanmail.net; 3 BK21 FOUR Team and Integrated Research Institute for Drug Development, College of Pharmacy, Dongguk University-Seoul, Goyang 10326, Korea; kaylee@dongguk.edu; 4 Gene Therapy Unit, KRIBB, Daejeon 34141, Korea; crjung@kribb.re.kr; 5 Laboratory Animal Resource Center, KRIBB, Chungbuk 28116, Korea; redbijou@kribb.re.kr; 6 Asan Medical Center, Department of Medical Science, Asan Medical Institute of Convergence Science and Technology, University of Ulsan College of Medicine, Seoul 05505, Korea; jung.kim0702@gmail.com (J.-A.K.); dbehdrn2992@naver.com (D.-G.Y.); aa0877@naver.com (E.-J.S.)

**Keywords:** LW6, hypoxia-inducible factor-1α, metabolite identification, hybrid triple quadrupole-linear ion trap mass spectrometer, predictive multiple reaction monitoring-information dependent acquisition-enhanced product ion

## Abstract

A novel HIF (hypoxia-inducible factor)-1α inhibitor, the (aryloxyacetylamino)benzoic acid derivative LW6, is an anticancer agent that inhibits the accumulation of HIF-1α. The aim of this study was to characterize and determine the structures of the metabolites of LW6 in ICR mice. Metabolite identification was performed using a predictive multiple reaction monitoring-information dependent acquisition-enhanced product ion (pMRM-IDA-EPI) method in negative ion mode on a hybrid triple quadrupole-linear ion trap mass spectrometer (QTRAP). A total of 12 metabolites were characterized based on their MS/MS spectra, and the retention times were compared with those of the parent compound. The metabolites were divided into five structural classes based on biotransformation reactions: amide hydrolysis, ester hydrolysis, mono-oxidation, glucuronidation, and a combination of these reactions. From this study, 2-(4-((3r,5r,7r)-adamantan-1-yl)phenoxy)acetic acid (APA, M7), the metabolite produced via amide hydrolysis, was found to be a major circulating metabolite of LW6 in mice. The results of this study can be used to improve the pharmacokinetic profile by lowering the clearance and increasing the exposure relative to LW6.

## 1. Introduction

Hypoxia-inducible factor (HIF)-1 is activated in response to intracellular hypoxia to cause genetic alterations that activate oncogenes and inactivate tumor suppressor genes [1,2,3]. HIF-1 consists of a constitutively expressed HIF-1β and one of three subunits (HIF-1α, HIF-2α, and HIF-3α) [4]. HIF-1α is the central transcriptional regulator of genes involved in the angiogenesis, metastasis, and therapy resistance of tumor cells. Thus, HIF-1α is considered as an attractive target for the development of new anticancer therapies [5,6]. In a previous study, a novel (aryloxyacetylamino)benzoic acid derivative, LW6, was reported to possess potential antitumor effects. It has been reported that LW6 promotes HIF-1α degradation by upregulating the von Hippel–Lindau tumor suppressor (VHL) [7].

Metabolite profiling studies of new chemical entities (NCEs) are widely performed in the pharmaceutical industry to support drug discovery [8,9]. These studies not only provide information about the metabolic fate of NCEs but also assist in identifying metabolic soft spots in the parent compound. Liquid chromatography-tandem mass spectrometry (LC-MS/MS) plays a key role as a powerful tool in the study of drug metabolism and pharmacokinetics (DMPK) owing to its sensitivity, selectivity, specificity, and speed [10,11,12]. It is used extensively for structural characterization of NCEs and their metabolites. The hybrid quadrupole linear ion trap mass spectrometer (QTRAP) is widely used to quantitate the parent drug and simultaneously screen metabolites in in vitro and in vivo samples [13,14,15]. LC-MS/MS analysis using independent data acquisition (IDA) with QTRAP facilitates simultaneous semi-quantification and structural confirmation of metabolites in a single sample injection. Predictive multiple reaction monitoring-information dependent acquisition-enhanced product ion mode (pMRM-IDA-EPI) is a useful method with higher sensitivity than other techniques [16,17]. The pMRM-IDA-EPI method is composed of one multiple reaction monitoring (MRM) scan, one IDA criterion, and one EPI scan. In the analysis, the MRM scan serves as a survey scan to trigger information-dependent acquisition of the EPI spectrum. This method can simultaneously provide quantitative and qualitative metabolite profiling information. This approach also can be helpful for identifying positional changes in metabolism.

In the current study, we analyzed the parent compound LW6 and its metabolites in mouse plasma samples using pMRM-IDA-EPI scan mode after intravenous or oral administration. The purpose of the present study was to establish a simple and practical strategy for analyzing and identifying LW6 and its metabolites. This practical strategy can be used to screen metabolites using in vivo samples.

## 2. Results

### 2.1. Fragmentation of LW6

**Figure 1:**
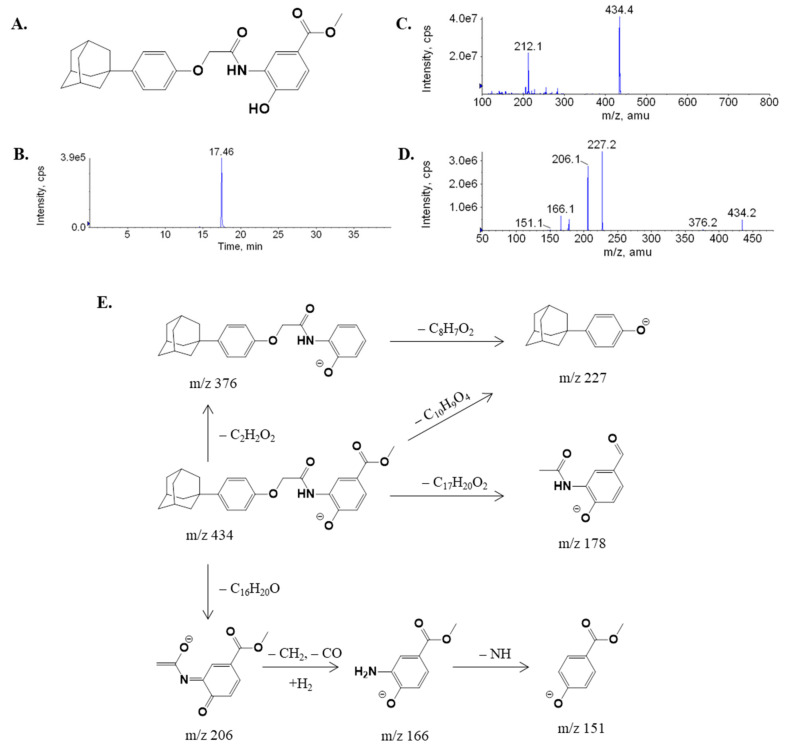
Structure (**A**), chromatogram (**B**), full-scan mass spectrum (**C**), enhanced product ion (EPI) spectrum at *m/z* 434 (**D**), and predominant fragmentation patterns for (aryloxyacetylamino)benzoic acid derivative (LW6) (**E**).

The first step in metabolite identification was to study the MS/MS fragmentation pattern of the parent compound LW6. The MS/MS fragmentation pattern of LW6 was examined in negative mode. A deprotonated molecular ion [M-H]^−^ of LW6 at *m/z* 434 was observed in the full-scan mass spectrum. The deprotonated molecules subsequently yielded a series of characteristic fragment ions in the QTRAP at *m/z* 376, 227, 206, 178, 166, and 151. LW6 was eluted at 17.46 min under the experimental conditions. The structure, representative chromatogram, MS/MS product ion spectrum, and predominant fragmentation patterns of LW6 are shown in Figure 1.

### 2.2. Metabolite Profiling of LW6 in Serial Mouse Plasma

To determine the in vivo metabolism of LW6, male ICR mice were administered a single intravenous (i.v., 5 mg/kg) or oral dose (p.o., 5 mg/kg) of LW6. Serial blood samples (Section 4.2) were collected and metabolite identification was performed using a pMRM-IDA-EPI scan method in negative ion mode on a QTRAP.

**Figure 2:**
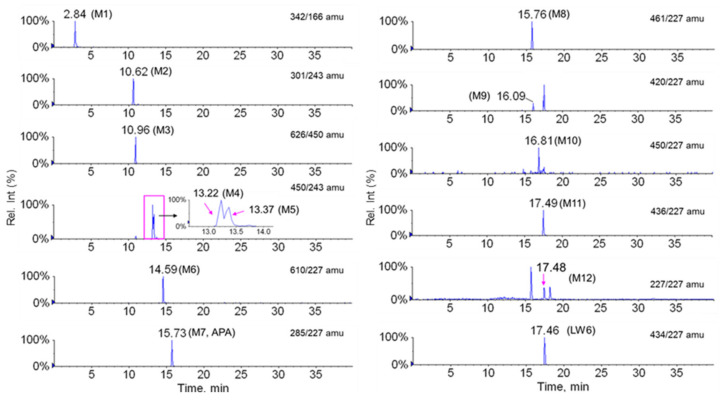
Extracted multiple reaction monitoring (MRM) chromatograms for LW6 and its metabolites from 5 mg/kg LW6-dosed mouse plasma samples.

A total of 12 metabolites were found in the mouse plasma samples, and their spectra were used for comparison with LW6 for structural confirmation (Figure 2). All 12 metabolites were detected in the mouse plasma obtained from the i.v. group. Eight of the metabolites (M1–M8) were detected in the mouse plasma obtained from the p.o. group.

**Figure 3:**
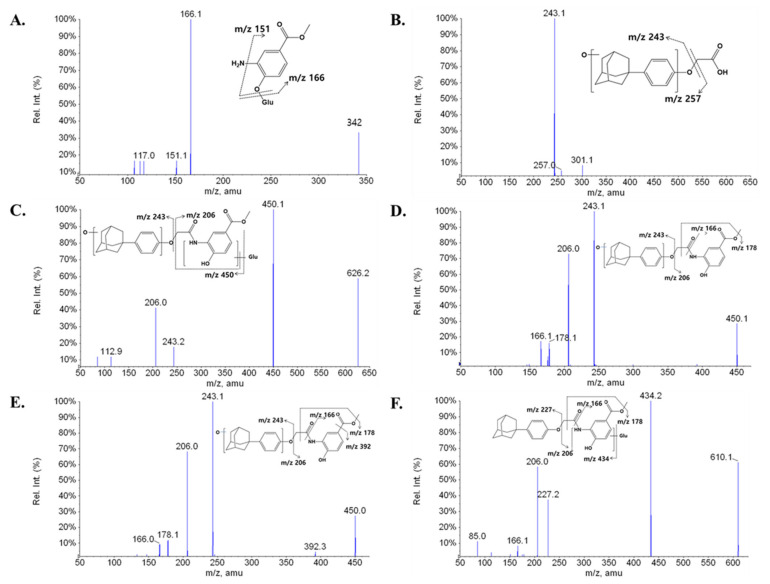

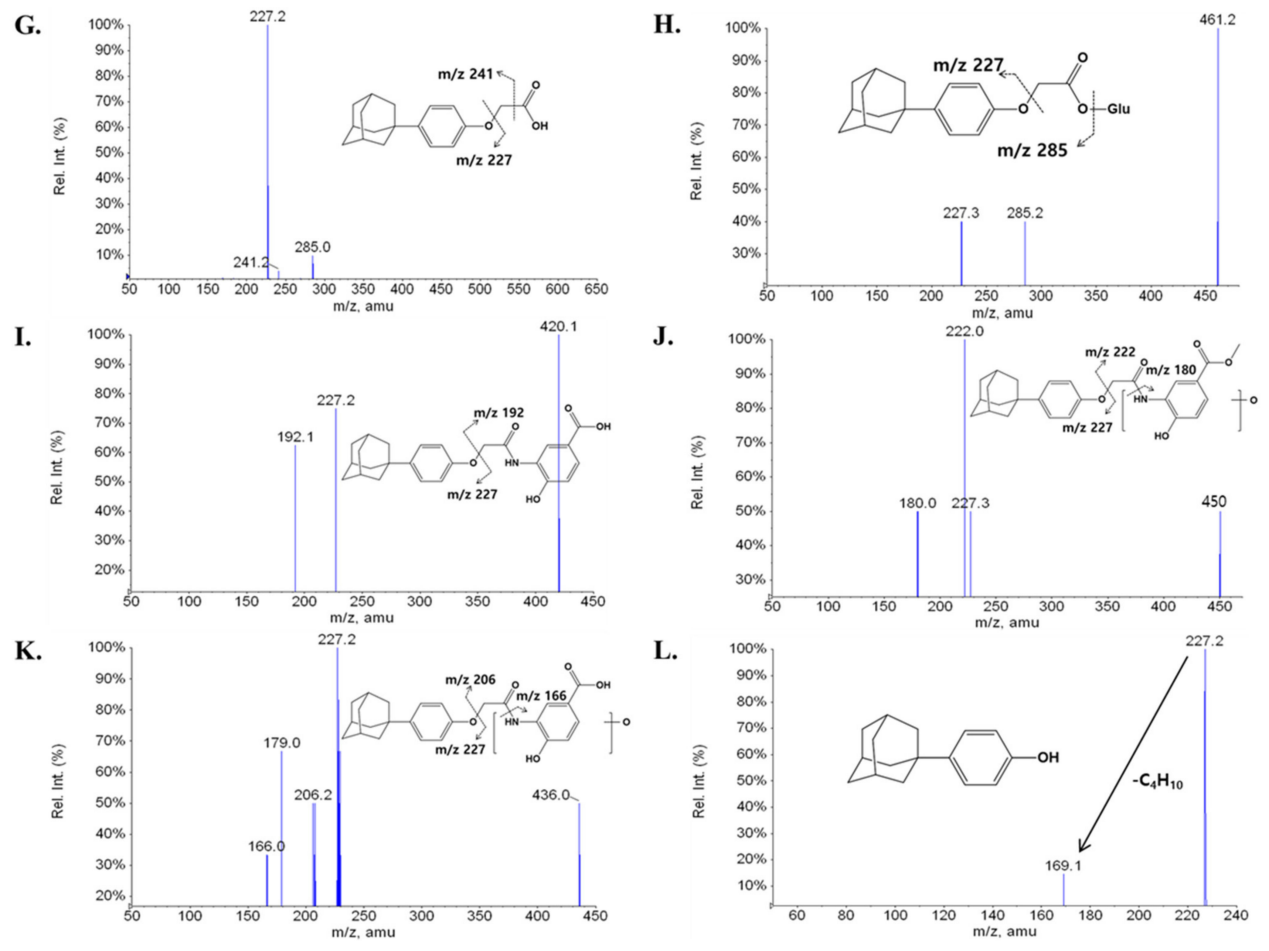
Enhanced product ion (EPI) spectra and proposed fragmentation patterns for the in vivo metabolites of LW6: M1 (**A**), M2 (**B**), M3 (**C**), M4 (**D**), M5 (**E**), M6 (**F**), M7 (**G**), M8 (**H**), M9 (**I**), M10 (**J**), M11 (**K**), and M12 (**L**).

The major metabolite of LW6, M7 (APA), was found to be a hydrolysis product (acid part after amide hydrolysis, a deprotonated molecule [M-H]^−^ at *m/z* 285) (Figure 3G). The structure of M7 was confirmed by comparing the spectrum with that of chemically synthesized APA (data not shown). The major MS/MS fragment ions of LW6, APA, and 11 proposed metabolites are summarized in Table 1. LC-MS/MS analysis of unchanged LW6 and its 12 metabolites produced informative and prominent product ions for structural determination (Figure 3).

The mass spectrum of M1, which was observed at a retention time 2.84 min, gave a deprotonated molecule [M-H]^−^ at *m/z* 342. The MS/MS spectrum of M1 showed a major fragment ion at *m/z* 166 (176 amu less than deprotonated M1) and a minor fragment ion at *m/z* 151 (loss of NH_2_) (Figure 3A). These results indicate that M1 is a glucuronide conjugate of product formed by amide hydrolysis (amine part) followed by glucuronidation.

M2 was eluted at a retention time of 10.62 min. M2 ([M-H]^−^ at *m/z* 301) was 16 amu larger than M7 ([M-H]^−^ at *m/z* 285). The MS/MS spectrum showed a series of characteristic product ions at *m/z* 257 and 243 (Figure 3B). The major fragment ion at *m/z* 243 exhibited a 16 amu difference compared with the fragment ion at *m/z* 227 of parent. This indicates that M2 is a formed by amide hydrolysis (acid part, M7) and mono-oxidation.

M3, which was observed at a retention time of 10.96 min, showed an [M-H]^−^ at *m/z* 626 and a fragment ion at *m/z* 450 (176 amu less than the [M-H]^−^) in the MS/MS spectrum, indicating that M3 contains a glucuronide group. Fragment ions at *m/z* 450 (loss of glucuronide), 243, and 206 were observed in the MS/MS spectrum of M3 (Figure 3C). These results suggest that M3 is a glucuronide conjugate of M4 or M5 formed by mono-oxidation.

Metabolites M4, M5, and M10 were observed at retention times of 13.22, 13.37, and 16.81 min, respectively, with the same deprotonated molecular ion observed at *m/z* 450. The three metabolites were 16 amu larger than LW6, suggesting hydroxylation of LW6 at structurally different sites. The characteristic product ions at *m/z* 166, 178, 206, and 243 were commonly observed in the MS/MS spectra of both M4 and M5 (Figure 3D,E). Fragment ions at *m/z* 166 and 206 were also observed in the MS/MS spectrum of LW6. The major fragment ion at *m/z* 243 was formed by oxidation of the adamantane ring or the phenolic ring. In contrast, a product ion at *m/z* 227 was observed in the MS/MS spectrum of M10, the same as for the parent. The most abundant fragment ion at *m/z* 222 exhibited a 16 amu difference compared with the fragment ion at *m/z* 206 of the parent (Figure 3J). This fragmentation pattern of M10 clearly indicates that the hydroxylation occurs on the right-hand side at the methoxycarbonyl phenolate.

M6 was detected at a retention time of 14.59 min with the deprotonated molecular ion at *m/z* 610. The ion at *m/z* 610 was increased by 176 amu compared with that of the parent, LW6. A series of characteristic product ions at *m/z* 434 (loss of glucuronide), 227, 206, 166, and 85 was found in the MS/MS spectrum of M6 (Figure 3F). Fragment ions at *m/z* 227, 206, and 166 were also observed in the MS/MS spectrum of LW6 (*m/z* 434). These results indicate that M6 is a glucuronide conjugate of the parent.

M8 was eluted at a retention time of 15.74 min. M8 ([M-H]^−^ at *m/z* 461) was 176 amu larger than M7 ([M-H]^−^ at *m/z* 285). The MS/MS spectrum showed a series of characteristic product ions at *m/z* 285 (loss of glucuronide) and 227 (Figure 3H). This suggests that M8 was formed by the glucuronide conjugate of M7.

M9, which exhibited a deprotonated molecular ion at *m/z* 420, was reduced by 14 amu compared with the parent. M9 was eluted at a retention time of 16.09 min, and the MS/MS peaks at *m/z* 227 and 192 were characteristic ions (Figure 3I). Therefore, M9 may have been produced by the elimination of a methyl group by ester hydrolysis on the right-hand side.

M11 was eluted at a retention time of 17.49 min. M11 ([M-H]^−^ at *m/z* 436) possessed 16 amu more than M9 ([M-H]^−^ at *m/z* 420). The MS/MS spectrum exhibited a series of characteristic product ions at *m/z* 227, 206, 179, and 166 (Figure 3K). The fragment ion at *m/z* 206 exhibited a 16 amu difference compared with the fragment ion at *m/z* 197 of M9. This indicates that M11 was formed by ester hydrolysis and mono-oxidation.

**Figure 4:**
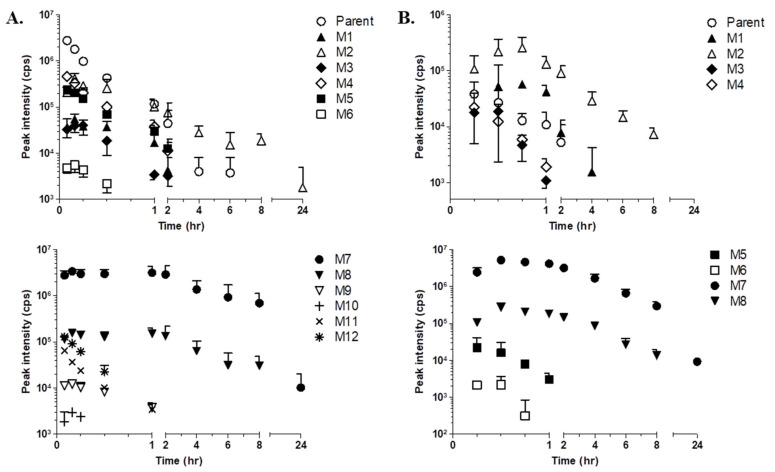
Peak area–time profiles for LW6 and its metabolites after intravenous (**A**) and oral (**B**) administration in mice. A single dose of LW6 was administered intravenously ((**A**); 5 mg/kg; n = 3) or orally ((**B**); 5 mg/kg; n = 3), and the plasma concentrations of LW6 and its metabolites were measured for 24 h post-dosing. Each point represents the mean ± S.D. (n = 3).

M12 was identified based on the MRM transition (*m/z* 227 > 227) because the predicted metabolite was equal to the transition of the selected product ion of the parent. M12 was detected at a retention time of 17.48 min. In this study, M12 may have been formed by O-dealkylation. The MS/MS spectrum showed product ions at *m/z* 227 and 169, possibly produced by the loss of C_4_H_10_ from the adamantane ring. Although structural information did not show definitive results, M12 appears to be an LW6-derived metabolite. M12 was not observed in the blank plasma but showed a time profile after i.v. administration (Figure 4).

### 2.3. Time Profiling and Relative Quantification of LW6 and its Metabolites

In the current study, kinetic analysis of the LW6 parent molecule and its metabolites was carried out simultaneously without authentic compounds for each metabolite. Figure 4 shows the peak area—time profiles of the metabolites tentatively identified from mouse plasma after i.v. or oral administration. The kinetic profiles indicate variation in the trends for LW6 and its metabolites. It is important to understand the kinetic profiles to determine the metabolic pathway more completely, particularly for metabolites mediated by or produced by consecutive reactions.

To estimate the relative systemic exposure of each metabolite formed from LW6, the area under the peak area—time curve (AUC) was calculated for each metabolite. The relative percentage of AUC values of each identified metabolite is summarized in Table 2. The relative comparison of each metabolite is also shown in Table 2.

The relative percentage of AUC of LW6 after p.o. administration was very low at only 2.7% of the LW6 AUC after the i.v. dose. APA (M7) was the most abundant metabolite resulting from both the i.v. and oral doses. The relative AUC of APA was much higher than the parent AUC by approximately 19-fold for the i.v. dose and 17-fold for the oral dose (Table 2). The relative percentage of APA as the sum of each metabolite’s AUC was calculated as 92.1% for i.v. and 93.3% for p.o. (Table 2). The metabolites with a relative percentage of the AUC of greater than 1% were ranked by relative abundance as follows: APA (M7, 92.1%) > M2 (3.1%) ≥ M8 (2.9 %) for i.v. dose, APA (M7, 93.3%) > M8 (3.7%) > M2 (2.4%) for p.o. dose.

## 3. Discussion

The main purpose of metabolite screening in early drug discovery is to identify metabolic soft spots that cause poor metabolic stability. This information can guide synthetic chemistry efforts by defining molecules with good pharmacokinetic properties. In the present study, we conducted metabolite profiling for LW6, which was selected as a lead candidate, by using the pMRM-IDA-EPI method with QTRAP.

A QTRAP is typically not suitable for full-scan screening because the detection sensitivity is rather poor compared with other types of MS instruments when full-scan acquisition is used. However, this instrument has MRM-EPI and multiple ion monitoring (MIM)-EPI scanning functions, which are not available on other types of mass spectrometers [18,19]. The MRM scan was used as a survey scan in this work because of its higher selectivity and sensitivity than EMS (Enhanced MS), NL (neutral loss), MIM, and PI (precursor ion) scans, and it can trigger an EPI scan to profile predicted metabolites in samples [19,20,21]. The main limitation of this method is its inability to detect unexpected metabolites that are not included in the expected metabolite list used to create the MRM transitions. The detection of metabolites by MRM is based on predicted molecular weights and fragmentation patterns. Therefore, unknown metabolites cannot be detected if neither the molecular weights nor the fragmentation patterns are predictable. However, MRM is suitable for multi-target screening. Moreover, the combination of MRM and EPI scans can be used to identify proposed metabolites, although MRM transitions are not sufficiently specific to determine metabolites in unknown samples without synthetic standards. For effective metabolite screening, predictive MRM should be designed by selecting appropriate theoretical metabolite ions and their product ions.

Recently, the U.S. Food and Drug Administration [22] and the International Conference on Harmonisation of Technical Requirements for Registration of Pharmaceuticals for Human Use [23] both issued formal regulatory guidelines on metabolites in safety testing (MIST) in February 2008 and June 2009, respectively. These guidelines emphasize the quantitative monitoring of metabolites in preclinical and clinical studies. They establish a strategy for toxicological evaluation of circulating metabolites. Most metabolite profiling studies have focused on qualitative analysis. Some studies have reported that quantitative MRM-IDA-EPI analysis was not affected by the addition of EPI scans for obtaining qualitative information during the same chromatographic run, compared with MRM-only methods [24,25]. In drug discovery, chemical synthesis of metabolite standards is often difficult and costly. Therefore, the MRM-IDA-EPI method is a useful approach for obtaining qualitative and semi-quantitative information about expected metabolites in a single LC-MS/MS analysis without authentic metabolite standards, although structural changes between a parent and metabolite molecule may cause dramatic differences in ionization efficiency.

Drug metabolism is classically divided into phase I and phase II reactions. Phase I reactions are oxidation, reduction, or hydrolysis of a parent drug, resulting in its conversion to a more polar metabolite(s). Phase II reactions include glucuronidation, sulfonation, acylation, methylation, and conjugation with glutathione or amino acids. In the current study, 33 MRM transitions were used to detect 24 expected metabolites. The possible metabolites were compiled based on metabolically vulnerable sites: aromatic ring (oxidation), -NH group (glucuronidation), ester bond (hydrolysis), amide bond (hydrolysis), *O*-alkyl group (*O*-dealkylation), and their combinations (Table 3). The present study demonstrated that LW6 was converted into at least 12 metabolites in mice (Table 1). M9, M10, M11, and M12 were only detected in plasma samples obtained from intravenous dosing. Our results also indicate that LW6 was mainly metabolized to APA (M7) (Table 2). M1, M2, M4, M5, M7, M9, M10, M11, and M12 were produced by phase I reaction, and M6 was produced by phase II metabolism, based on the structure information of its metabolites. M8 was produced through serial processes of phase I reaction and then phase II reaction. M3 was also the product of combination of phase I and II reactions, or reversely, phase II and I reactions. It was challenging to assign the specific metabolism pathway to each metabolite since phase I and II metabolism systems are not distinguishable in mice.

The relative importance of a metabolite compared with the parent compound as well as other metabolites can be predicted using the in vivo or in vitro metabolite kinetic profile [26]. To evaluate the relative abundance of each metabolite formed from LW6, the AUC of each metabolite was calculated from LC-MS/MS data. An important principle of metabolite kinetics is that the in vivo disposition of a metabolite depends on its formation and elimination. The measurement of the AUC for a metabolite in a time series reflects its formation and elimination. Our results show that APA is a major metabolite of LW6 in mice. Based on the AUC_0→t_ for LW6 administered intravenously, the AUC_0→t_ for the metabolite APA was highly increased by approximately 19- or 18-fold after LW6 was administrated intravenously or orally, respectively (Table 2). Although the AUC_0→t_ of LW6 was significantly different between the oral (2.7%) and intravenous (100%) administration routes, the AUC_0→t_ of the produced metabolite, APA, was very similar (Table 2). As shown in Table 2, the AUC_0→t_ of APA relative to the sum of the AUC_0→t_ for all detected metabolites is 92% (i.v.) or 93% (p.o.). These results indicate that rapid disappearance of the parent LW6 in plasma after an oral dose could be due to rapid conversion to APA by hydrolysis of LW6 in mice.

**Figure 5:**
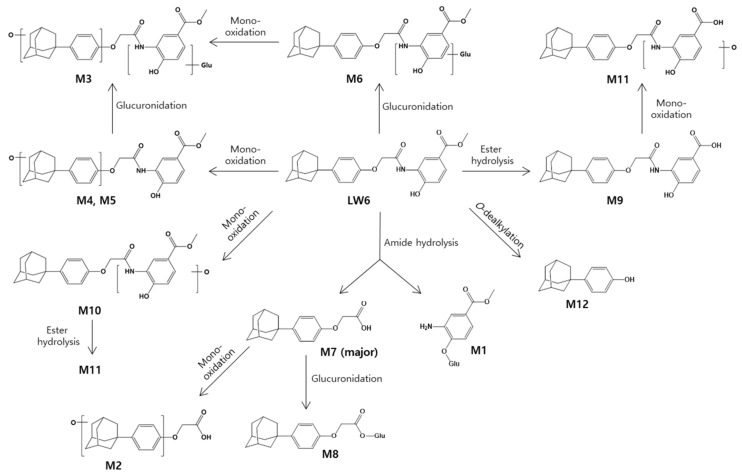
Proposed metabolic pathways of LW6 in mice.

The time-course analysis of the metabolites of LW6 provided information about the primary and secondary metabolites. This approach helps to eliminate the false identification of metabolites and to understand the metabolite kinetics. Thus, this method provides a rationale for the proposed metabolic pathway because the data show the kinetic information for metabolite formation and disappearance. In the present study, LW6 was rapidly and predominantly converted to APA (M7). The formation and elimination of M2 and M8 paralleled that of APA (M7). LW6 is thought to form APA relatively rapidly in mice, indicating that the secondary metabolite formed from LW6 through hydroxylation or glucuronidation with APA. The proposed metabolite structures and possible metabolic pathways for LW6 are shown in Figure 5.

In summary, LC-MS/MS analysis using the MRM-IDA-EPI approach on a hybrid quadrupole mass spectrometer can be used to support simultaneous quantitative and qualitative analysis in metabolite profiling studies. In this study, MRM scans were used as survey scans owing to their higher selectivity and sensitivity than EMS, NL, MIM, and PI scans. EPI spectra from the parent and potential metabolites were obtained within the same chromatographic run, and these spectra were used to confirm the proposed metabolite structures. Our results demonstrate that LW6, a novel HIF-1α inhibitor, was rapidly hydrolyzed to its major metabolite, APA (M7), in mice after both intravenous and oral administration. APA (M7) was also further metabolized to M2 by hydroxylation or M8 by glucuronidation.

## 4. Materials and Methods

### 4.1. Chemicals and Reagents

Acetonitrile (HPLC grade) was purchased from Thermo Fisher Scientific Co. (Waltham, MA, USA). Formic acid (HPLC grade), dimethylacetamide, Cremophor EL, (2-hydroxypropyl)-β-cyclodextrin, and 4-methylumbelliferone were purchased from Sigma-Aldrich (St. Louis, MO, USA). All other chemicals were of the highest quality available. LW6 (> 96.04% purity) and 2-(4-((3r,5r,7r)-adamantan-1-yl)phenoxy)acetic acid (APA, > 99.99% purity) were provided by Dr. Kyeong Lee at the College of Pharmacy, Dongguk University (Goyang, Korea). Microvette^®^ capillary tubes were purchased from Sarstedt (Nümbrecht, Germany).

### 4.2. Animal Study

Animal experiments were approved by the Institutional Animal Care and Use Committee of the Korea Research Institute of Bioscience and Biotechnology (Chungbuk, Korea; KRIBB-AEC-15005, 9 January 2015) and performed in compliance with the National Institutes of Health Guidelines for the care and use of laboratory animals and Korean national laws for ethical conduct in the care and use of animals and the rules of Good Laboratory Practice. Six specific pathogen-free (SPF) male ICR mice (8 weeks, 31.5–40.1 g) were purchased from Koatech Co. (Pyeongtaek, Kyonggi, Korea) and maintained in an SPF environment at 22 ± 2 °C with a 12 h light/dark cycle and relative humidity of 50 ± 10%. Food (Teklad Global rodent diet 2018S, ENVIGO, Indianapolis, IN, USA) and water were provided ad libitum, unless noted otherwise. Animals were acclimated to the testing facility for 1 week before being used in the study. The animal group for oral dosing was fasted overnight before the study and fed again 4 h after dosing. Mice were administered a single dose of the test substance intravenously via tail vein injection (n = 3, 5 mg/kg) or orally by disposable syringe with an oral zonde (n = 3, 5 mg/kg). Dosing solutions were prepared in dimethylacetamide/Cremophor EL/20% *v/v* (2-hydroxypropyl)-β-cyclodextrin in de-ionized water (1/1/3 *v/v/v*; i.v. or p.o. for LW6) and administered at a dose volume of 5 mL/kg for both i.v. and p.o. Peng et al. method was used for serial blood sampling in mice [27]. Briefly, blood samples (~50 µL each) were collected via the saphenous vein of free alive mice at the time points including 0, 0.25, 0.5, 0.75, 1, 2, 4, 6, 8, and 24 h after oral administration or 0, 0.083, 0.167, 0.25, 0.5, 1, 2, 4, 6, 8, and 24 h after intravenous administration. These samples were collected into Microvette^®^ capillary tubes at predetermined time points. The blood samples were centrifuged under refrigeration at 12,000× *g* for 3 min and then stored frozen at −20 °C until analysis.

### 4.3. Sample Preparation

Plasma samples were prepared for analysis by the protein precipitation method [28]. A simple protein precipitation is to remove proteins in plasma samples with organic solvents. Briefly, 15 µL of each plasma sample was transferred to a PCR tube (Axygen, Union City, CA, USA). Four volumes of acetonitrile containing the analytical internal standard, 4-methylumbelliferone, were added, and the resulting mixture was vortexed for 10 min on a multi-tube vortexer (VWR International, Radnor, PA, USA). The samples were sonicated for 30 min at room temperature. The tubes were centrifuged at 12,000× *g* for 10 min, and the supernatant was analyzed for the test substance. Sample analysis was performed with a 3200 Q TRAP LC/MS/MS system (Applied Biosystems, Foster City, CA, USA) in negative MRM mode. The analytical methods are described in the following sections.

### 4.4. Chromatographic and Mass Spectrometric Methods

The LC-MS/MS system consisted of an Agilent 1100 series HPLC system (Agilent Technologies, Santa Clara, CA, USA) equipped with an API 3200 QTRAP hybrid triple quadrupole-linear ion trap mass spectrometer with a Turbo V^TM^ ion spray. All samples were analyzed on an Acquisition mode and data were processed using Analyst^®^ software (version 1.4.2, Applied Biosystems, Foster City, CA, USA). The sample injection volume was 10 µL, and separation was performed using an Atlantis dC18 column (50 × 2.1 mm i.d., 3 µm, Waters, Milford, MA, USA) with a SecurityGuard^TM^ C18 guard column (2.0 × 4.0 mm i.d., Phenomenex, Torrance, CA, USA) maintained at room temperature. The column was preequilibrated with 95% *v/v* solvent A (deionized water containing 0.5% *v/v* acetic acid)/5% *v/v* solvent B (acetonitrile containing 0.5% *v/v* acetic acid) at a flow rate of 0.4 mL/min. A linear gradient of the two solvents was used. Initially, 5% B was linearly increased to 50% B over 10 min, increased to 80% B over 6 min, kept constant at 80% B for 4 min, linearly increased to 95% B over 10 min, kept constant at 95% for 10 min, and switched back to the initial conditions. The flow rate was set to 0.35 mL/min throughout the gradient. To optimize the source parameters, an LW6 standard was used to optimize the major mass parameters, such as declustering potential (DP) and collision energy (CE). Product ions of *m/z* 227 or 166 were selected to search for new metabolites. Each transition was monitored with a 30 ms dwell time. The total cycle time was approximately 1.61 s. The instrument was operated in negative ion electrospray mode at an ion spray voltage of −4500 V, the source temperature was maintained at 600 °C, and the collision gas was set to high. The nebulizer gas (NEB), curtain gas (CUR), and collision gas (CAD) were set to 40 psi, 20 psi, and high, respectively. Nitrogen gas was used for CUR, CAD, and NEB. The interface heater was on.

### 4.5. Predicted MRM-Enhanced Product Ion Scan (pMRM-EPI) Method

The pMRM-EPI method was used in this study. Specific MRM information for the predicted metabolites was conducted with Microsoft Excel using the measured optimal MRM information for the parent compound, LW6, and the predicted mass changes for the metabolites. For every metabolite, one or two specific MRM functions were generally calculated based on the precursor and selected product ion of LW6, as listed in Table 3. For the MRM function of the predicted metabolite, the original mass of the product ion was used, or the difference corresponding to the metabolic transformation was incorporated. If the mass of the predicted metabolite was equal to or smaller than that of the selected product ion from the parent, a multiple ion monitoring (MIM)-EPI experiment was carried out in MRM-EPI mode. Unlike the MRM scan, the MIM scan was done with a minimal CE (5 eV) in Q2 so that the metabolite isolated in Q1 passed through Q2 with minimal fragmentation. Thus, the same ions were monitored in Q1 and Q3. A total of 33 MRM transitions including MIM (5 transitions) and MRM (28 transitions) were employed in the survey experiment. The MS/MS spectra for the metabolites can be obtained using the IDA of the EPI. The IDA threshold was set to 500 counts per second (cps) for the MIM and MRM scans. EPI scans were performed in profile mode with a fixed LIT (linear ion trap) fill time of 20 ms, a step size of 0.12 amu, and an ion scan range of 50–650 amu with a scan rate of 4000 amu/s. The CE was set to −40 eV with a CE spread of 10 eV.

### 4.6. Data Processing Method and Set Criteria for Metabolite Hits

The actual samples were compared with the 0 min samples without LW6. Positive hits were also compared manually with the 0 min samples (pre-dose samples) to distinguish them from possible impurities present with LW6. A detectable peak (S/N ratio > 3) in the samples that was not present in the 0 min samples was considered as a positive hit. The peak detection threshold for absolute peak area was set to slightly overcome the normal instrument noise. All MRM-EPI data were processed manually. The MRM-EPI acquisition generated two sets of LC/MS data, expressed as the total MRM and MS/MS ion chromatograms. The total MRM ion chromatogram shows all of the ions detected by the MRM scan. The total MS/MS ion chromatogram contains all the MS/MS spectra acquired by the MRM-directed EPI scan. Both the MRM and EPI data sets were processed by extracted ion chromatogram (XIC) analysis. The results are expressed as processed or extracted MS/MS or MRM chromatograms. Analyst 1.4.2 software (Applied Biosystems) was used for data processing with XIC.

### 4.7. Semi-Quantitation of Targeted Metabolites

To evaluate the relative abundance of each LW6 metabolite, the AUC of each metabolite was determined relative to that of the parent drug. The areas under the peak intensity–time curves (AUC_0__→t_) were calculated by the linear-trapezoidal method using Kinetica^TM^ 4.4.1 (Thermo Fisher Scientific).

## Figures and Tables

**Table 1:** Major MS/MS fragments of LW6 and its metabolites.

Metabolite	Retention Time (min)	[M-H]^−^	Major Fragments
LW6 (parent)	17.46	434	**227**, 206,166
APA (M7)	15.73	285	241, **227**
M1	2.84	342	**166**, 151, 117
M2	10.62	301	257, **243**
M3	10.96	626	**450**, 243, 206, 113, 85
M4	13.22	450	**243**, 206, 178, 166
M5	13.37	450	392, **243**, 206, 178, 166
M6	14.59	610	434, **227**, 206, 178, 166, 85
M8	15.76	461	285, **227**
M9	16.09	420	**227**, 192
M10	16.81	450	**227**, 222, 180
M11	17.49	436	**227**, 206, 179, 166
M12	17.48	227	**227**, 169

Bold ions reflect the selected daughter ion for MRM mode.

**Table 2:** Relative percentage of area under the peak area–time curve (AUC) values for LW6 and its metabolites.

Metabolite	i.v.			p.o.		
	Average AUC_0__→t_ (Peak Intensity, Cps)	Relative % of Parent AUC_0__→t_ (i.v.) ^1^	Relative % of Total Metabolite AUC_0__→t_^2^	Average AUC_0__→t_ (Peak inTensity, cps)	Relative % of Parent AUC_0__→t_ (p.o.) ^1^	Relative % of Total Metabolite AUC_0__→t_^2^
LW6 (Parent)	1,048,883	100.00	-	28,735	2.74	-
APA (M7)	19,697,150	1877.92	92.09	17,688,080	1686.37	93.32
M1	42,982	4.10	0.20	66,545	6.34	0.35
M2	653,800	62.33	3.06	457,710	43.64	2.41
M3	23,904	2.28	0.11	10,404	0.99	0.05
M4	170,950	16.30	0.80	10,340	0.99	0.05
M5	116,583	11.11	0.55	11,845	1.13	0.06
M6	2445	0.23	0.01	1127	0.11	0.01
M8	622,040	59.30	2.91	708,375	67.54	3.74
M9	7672	0.73	0.04	-	-	-
M10	511	0.05	0.00	-	-	-
M11	17,015	1.62	0.08	-	-	-
M12	31,345	2.99	0.15	-	-	-

^1^ The relative percentage of AUC values represents the relative AUC_0__→t_ for LW6 or each metabolite to the AUC_0__→t_ of the parent after intravenous administration (5 mg/kg). ^2^ The relative percentage of AUC values represents the relative AUC_0__→t_ of each metabolite to the sum of the AUC_0__→t_ of all the detected metabolites.

**Table 3:** List of biotransformations for profiling of LW6 and its metabolites in mice.

Type of Biotransformation	[M-H]^−^ *m/z*	pMRM
Parent (LW6)	434	434 > 227
Mono-oxidation	450	450 > 227, 450 > 243
Di-oxidation	466	466 > 227, 466 > 243
Glucuronidation	610	610 > 227, 610 > 243
Mono-oxidation + glucuronidation	626	626 > 450, 626 > 243
Ester hydrolysis	420	420 > 227
Ester hydrolysis + mono-oxidation	436	436 > 227, 436 > 243
Ester hydrolysis + mono-oxidation + glucuronidation	612	612 > 227, 612 > 243
Ester hydrolysis + glucuronidation	596	596 > 420, 596 > 227
Amide hydrolysis (amine part)	166	166 > 166
Amide hydrolysis + ester hydrolysis	152	152 > 152
Amide hydrolysis + glucuronidation	342	342 > 166
Amide hydrolysis + ester hydrolysis + glucuronidation	328	328 > 152
Amide hydrolysis (acid part)	285	285 > 227
Amide hydrolysis + glucuronidation	461	461 > 227
Amide hydrolysis + mono-oxidation	301	301 > 243
Amide hydrolysis + mono-oxidation + glucuronidation	477	477 > 301, 477 > 243
*O*-dealkylation (alcohol part)	227	227 > 227
*O*-dealkylation + glucuronidation	403	403 > 227
*O*-dealkylation + mono-oxidation	243	243 > 243
*O*-dealkylation + mono-oxidation + glucuronidation	419	419 > 243
*O*-dealkylation (aldehyde part)	222	222 > 222
*O*-dealkylation + glucuronidation	398	398 > 222
*O*-dealkylation + aldehyde oxidation	238	238 > 238
*O*-dealkylation + aldehyde oxidation + glucuronidation	414	414 > 238

## Data Availability

The data presented in this study are available on request from the corresponding author.

## Sample Availability

Samples of the compounds presented in the paper are available on request from the corresponding author.
